# Hippocampal Glutamatergic Hyperactivation Mediates High‐Loading Intensity of Exercise‐Induced Cognitive Deficits Via HPC‐mPFC Circuit Dysfunction

**DOI:** 10.1002/cns.70928

**Published:** 2026-06-18

**Authors:** Qian Bai, Le Wang, Hedong Lang, Hongtao Yu, Xiaolei Wang, Jian Wang, Jundong Zhu, Ka Chen, Mantian Mi

**Affiliations:** ^1^ Chongqing Key Laboratory of Nutrition and Health, Research Center for Nutrition and Food Safety, Chongqing Medical Nutrition Research Center, Institute of Military Preventive Medicine Army Medical University (Third Military Medical University) Chongqing China; ^2^ Department of Nutrition, Xinqiao Hospital Army Medical University (Third Military Medical University) Chongqing China

**Keywords:** chemogenetics, cognitive function, glutamatergic neurons, high‐loading intensity of exercise, hippocampus

## Abstract

**Aims:**

Excessive exercise impairs cognition and elevates late‐life cognitive risk. However, the underlying mechanisms remain unclear. This study investigates the neural mechanisms through which high‐intensity endurance exercise induces cognitive deficits.

**Methods:**

Mice underwent high‐loading intensity exercise (HLIE) with a 7‐day treadmill procedure (25 m/min, 90 min/day). A battery of behavioral tests was conducted to assess cognitive performance, including the Morris Water Maze, Novel Object Recognition, and Y Maze. fMRI and c‐Fos activity mapping were employed to identify key brain regions affected by HLIE. Single‐nucleus RNA sequencing (snRNA‐seq) was conducted to analyze transcriptional changes associated with disrupted neural activity. We used chemogenetic inhibition to identify the role of hippocampal glutamatergic neurons in HLIE‐induced cognitive deficits.

**Results:**

HLIE caused significant spatial and working memory deficits. The hippocampus (HPC) was the primary brain region affected, exhibiting reduced functional connectivity with the medial prefrontal cortex (mPFC) and disrupted transcriptional profiles linked to neural activity. Further, hippocampal glutamatergic neurons were particularly activated by HLIE, and chemogenetic inhibition prevented cognitive function following HLIE exposure.

**Conclusion:**

HLIE impairs cognition via hippocampal glutamatergic neuronal hyperactivation and downstream HPC‐mPFC circuit dysfunction. Our findings identify neuronal calcium dysregulation as a targetable mechanism for preserving cognition in high‐intensity exercise paradigms.

## Graphical abstract

High‐loading intensity of exercise (HLIE) disrupts HPC‐mPFC connectivity and hippocampal activity‐related transcriptomes, mediating cognitive deficits via glutamatergic neuron hyperactivation. Hippocampal glutamatergic neuron hyperactivation drives HLIE‐induced cognitive deficits, while targeted chemogenetic inhibition partly rescues cognitive function.

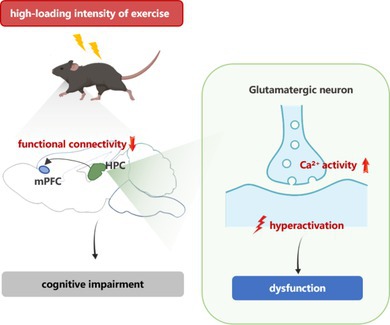

## 1 Introduction

Moderate aerobic exercise, as recommended by the World Health Organization (WHO), improves overall health [1]. However, in real‐world scenarios, certain groups, such as elite athletes and military personnel, often engage in excessive exercise characterized by high intensity and prolonged duration, which refers to ≥ 6 metabolic equivalents (METs) or ≥ 64% maximal oxygen uptake (VO_2max_) of substantial duration (e.g., ≥ 1 h) [2, 3, 4]. The impact of excessive exercise on health has garnered significant attention from scholars, particularly its negative effects on the heart, skeletal muscles, and the immune system, which have been well‐documented [5, 6, 7]. Despite limited evidence, a systematic review indicated overtraining results in cognitive decline [8], correlating with elevated dementia risk in later life [9, 10, 11]. Such impairments can, in turn, reduce physical performance, cognitive control, arousal, and alertness [12, 13]. However, the mechanisms remain poorly understood.

Cognitive processes rely on integrated brain networks involving the hippocampus (HPC), amygdala, and various cortical areas [14, 15, 16]. Notably, dysregulation in these cognitive‐related regions have been observed in individuals with excessive exercise or exercise‐induced fatigue [17, 18, 19]. Rodent models demonstrate exercise‐induced memory deficits associated with hyperactivation of neuroinflammation, impaired synaptic plasticity, and disrupted mitochondrial energetics in both the HPC and PFC [13, 20]. Numerous studies have reported a direct projection from the HPC to the medial prefrontal cortex (mPFC), a connection that regulates cognition and is susceptible to stress [21, 22, 23, 24, 25]. Abnormalities in the structure and connectivity of the HPC‐mPFC circuit are frequently observed during pathologic progression of neurodegenerative diseases, often leading to varying degrees of cognitive dysfunction [26, 27], yet its role in exercise‐driven deficits requires validation.

Comprehensive analyses of the molecular identity of neural cell types have provided important insights into understanding neurobehaviors under both physiological and pathological conditions [28, 29, 30]. Therefore, it is of great significance to analyze the impact of different neuronal subpopulations in specific regions on cognitive impairment induced by intense exercise. Notably, the projections of the HPC‐mPFC circuit are primarily glutamatergic [31], its excitation/inhibition imbalance drives cognitive decline in neurological disorders [32, 33]. Thus, the related neurobiological changes in excessive exercise‐induced impairment warrants investigation.

In the present study, we conducted behavioral tests, fMRI and c‐Fos staining to determine the effect of HLIE on cognitive performance and to investigate the underlying mechanisms. Then, we explored alterations in the transcriptional profiles of the HPC using snRNA‐seq analysis. Additionally, we conducted fiber photometry recordings, chemogenetic manipulation combined with behavioral tests to identify the specific neuronal populations contributing to cognitive impairment following HLIE. These findings will help elucidate the neural mechanisms underlying cognitive deficits related to HLIE.

## 2 Materials and Methods

### 2.1 Animals and Experimental Design

**FIGURE 1:**
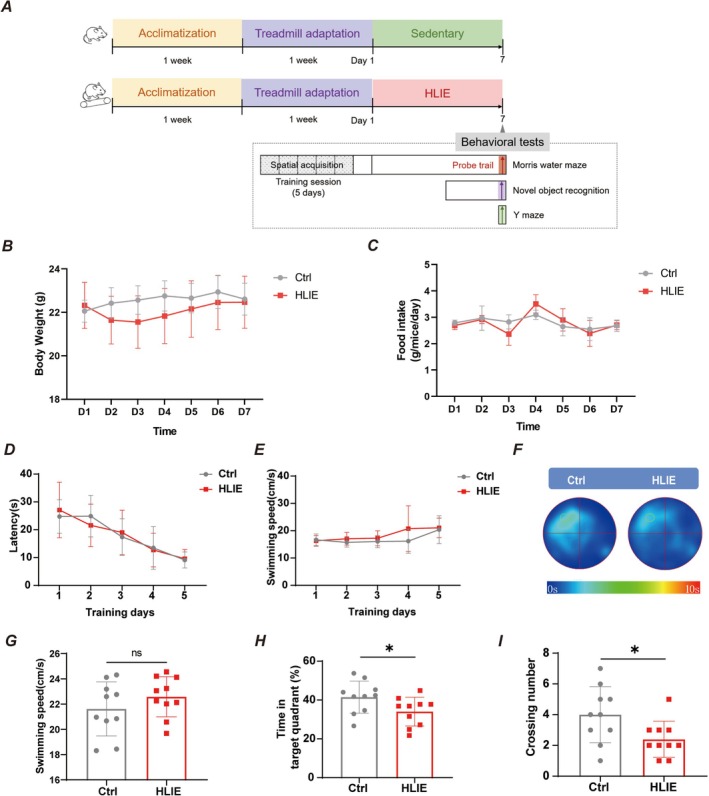
Impaired spatial memory performance in MWM of mice after 7‐day HLIE exposure. (A) Schematic of experimental design and the timeline of behavioral tests. (B, C) Changes of body weight (B) and food intake (C) of the two groups during the HLIE session. (D, E) There was no significant difference in the escape latency (D) or swimming speed (E) between HLIE and Ctrl mice over MWM training session. Significance was assessed by two‐way repeated‐measures analysis of variance (ANOVA) with post hoc comparisons between groups. (F) Heatmaps of swimming paths during the probe trials. (G–I) Swimming speed (G), the percentage of time spent in the target quadrant (H), and crossing number (I) of the target quadrant during the probe trials. *n* = 10 mice per group. Data are presented as mean ± SD. *p < 0.05; ns, no significance.

Male C57BL/6 mice (8 weeks old, 18.3–23.0 g) were purchased from Laibite Biotechnology Co. Ltd. (Chongqing, China) and kept at a constant temperature (22°C ± 2°C) and humidity (50%–55%) in a controlled facility on a 12 h light/dark cycle with standard food (D12450B; 10% fat, 70% carbohydrate, 20% protein) and water available ad libitum. After 7‐day acclimation, mice were randomly divided into two groups: the control group (Ctrl) and the HLIE group. The exercise procedure was based on Bedford's method [34, 35] and our previously reported procedure [36]. The treadmill protocol was selected based on established rodents' metabolic benchmarks. Previous evidence confirms treadmill exercise (> 20 m/min at ≥ 0% slope) should be identified as high intensity [37]. Meanwhile, the exercise regimen of our study elicits 73.80% ± 7.46% VO_2_ max in 8‐week‐old male Wistar rats [38], aligning with VO_2_ max was almost reached at running speeds of 25 m/min for C57BL/6J adult mice [35]. This range exceeds the 64% VO_2_max threshold defining vigorous intensity according to ACSM stratification criteria [4]. To brief, all mice were placed on a motorized treadmill (SANS Biological Technology, China) with a 10° incline for 10 min of running each morning at a speed of 15 m/min for 5 days as a pre‐adaptation phase. After 2 days of rest, mice in the HLIE group ran on the same treadmill at a speed of 25 m/min for 90 min per day for 7 days, while mice in the Ctrl group kept sedentary on the treadmill. A battery of behavioral tests was conducted after HLIE, including Morris water maze (MWM) [39], novel object recognition (NOR) [40, 41] and Y maze [42], as previous studies described. Detailed protocols are in Method and Materiel S1. The experimental design is shown in Figure 1A.

### 2.2 Magnetic Resonance Imaging Data Acquisition and Statistical Analysis

Within 2 h after the completion of 7‐day HLIE protocol, resting‐state functional MRI (rs‐fMRI) images were acquired from mice (Ctrl group, *n* = 3; HLIE group, *n* = 3) using a 7.0 T small animal Bruker scanner (PharmaScan, Germany). Mice were anesthetized with 5% isoflurane/oxygen and maintained under 2% isoflurane/oxygen during the scan. Respiration rate, heart rate, and body temperature were continuously monitored to ensure animal welfare and stable physiological conditions during imaging. Anatomical images were acquired using a rapid acquisition with refocused echoes (RARE) sequence with the following parameters: image size = 256 × 256, slice thickness = 0.6 mm, TE = 35.00 ms, TR = 3500.00 ms, averages = 6, echo spacing = 11.667 ms, repetitions = 1, field of view (FOV) = 20 mm × 20 mm, RARE factor = 8. Functional scans were acquired using a gradient‐echo echo planar imaging (EPI) sequence with the following parameters: image size = 96 × 96, slice thickness = 0.6 mm, TE = 18.649 ms, TR = 2000.00 ms, averages = 1, repetitions = 300, band width = 156250.0 Hz, field of view (FOV) = 20 mm × 20 mm. Image preprocessing, functional connectivity values, and power spectrum analysis were using SPM12 and MATLAB software by Siying Technology (Siying Technology Co. Ltd., Nanjing, China). 2D masks were drawn within the respective anatomically defined regions of interest (ROIs), where voxel thresholds (voxels > 10) and a significance level of *p* < 0.005 were applied.

### 2.3 Tissue Collection

After HLIE regimen, mice were deeply anesthetized with 1% sodium pentobarbital (100 mg/kg, i.p) and transcardially perfused with ice‐cold PBS, followed by ice‐cold 4% paraformaldehyde (PFA) (AR1069, BOSTER, China). Brains were rapidly extracted and post‐fixed in 4% PFA at 4°C overnight for subsequent staining procedures. Mouse serum was obtained by collecting blood from the orbital sinus, and regional brain tissues were dissected on ice and stored at −80°C until further use. For snRNA‐seq analysis, HPC tissues were quickly dissected and snap‐frozen in liquid nitrogen.

### 2.4 Biochemical Analysis

Urea, lactate dehydrogenase (LDH), and creatine kinase (CK) in the serum were determined using Urea Assay Kit (URE01X, Purebio, China), Lactate Dehydrogenase Assay Kit (LDH01X, Purebio, China), and Creatine Kinase Assay Kit (CK01X, Purebio, China), respectively, following the manufacturer's instructions. Measurements were performed using an automatic biochemical analyzer (2110, Hitachi, Japan).

### 2.5 snRNA‐Seq Analysis

snRNA‐seq was performed on HPC tissues (*n* = 3/group) using 10× Genomics Chromium Single Cell Ranger v7.0. Detailed protocols are in Method and Materiel S2.

### 2.6 Immunofluorescence Staining and Imaging

Brains were sectioned coronally at 30 μm using a cryostat (LEICA CM1950, Leica, Germany). Brain sections were blocked with 5% BSA (ST023, Beyotime, China) and 0.2% TritonX‐100 (ST795, Beyotime, China) in PBS for 2 h at room temperature (RT). The sections were then incubated with primary antibodies: anti‐NeuN (1:1000; 104224, Abcam), anti‐c‐Fos (1:1000; ABE457, Millipore), at 4°C overnight followed by probing with corresponding secondary antibody: goat anti‐mouse IgG (H + L) (1:1000; 4408, Cell Signaling Technology), goat anti‐rabbit IgG (H + L) (1:1000; 4414, Cell Signaling Technology), for 90 min at RT. The sections were then counterstained with DAPI (C1006, Beyotime) for 5 min to label nuclei. Finally, the sections were mounted on gelatin‐coated slides and coverslipped with Fluoromount‐G mounting medium (0100‐01, Southernbiotech, USA). Fluorescent images were acquired using the Olympus VS120 virtual microscopy slide scanning system (Olympus, Japan).

### 2.7 Stereotaxic Surgery and Viral Injection

Mice were deeply anesthetized with 1% sodium pentobarbital (100 mg/kg, i.p) and positioned in a stereotaxic frame (68018, RWD). Eye ointment was applied to prevent corneal desiccation, and body temperature was maintained at 37°C using a heating pad. After fur removal using depilatory cream and skin sterilization with 75% ethanol, a single midline anteroposterior scalp incision was made to expose the skull and a burr hole was drilled above the target region. For specific labeling of cellular Ca^2+^ in glutamatergic neurons of the HPC (AP: −2.0 mm, ML: −1.5 mm, DV: −1.5 mm), AAV expressing CaMKIIa‐GCaMP6f (rAAV9‐CaMKIIa‐GCaMP6s‐WPRE‐hGH polyA, PT0110, BrainVTA) was injected. For chemogenetic inhibition of glutamatergic neurons in the HPC, an AAV expressing the DREADD vector hM4Di was injected bilaterally into the HPC (AP: −2.0 mm, ML: ±1.5 mm, DV: −1.5 mm): rAAV9‐CaMKIIa‐hM4D(Gi)‐EGFP‐WPRE‐pA (PT0524, BrainVTA). For control experiments, an AAV expressing the control vector rAAV9‐CaMKIIa‐EGFP‐WPRE‐hGH polyA (PT0290, BrainVTA) was injected. AAV (300 nL) was injected at a rate of 30 nL/min using a pulled glass pipette, which was left in place for 10 min after injection to allow viral diffusion. After injection, the scalp was sutured and cleaned with antiseptic. Mice were monitored and kept warm until fully recovered before being returned to their cages. Mice were subjected to HLIE protocol and administered clozapine N‐oxide (CNO) injection (i.p., 5 mg/kg). After completing the HLIE protocol, a novel object recognition test was performed to assess cognition.

### 2.8 Fiber Photometry Recordings

Three weeks after fiber implantation, mice were subjected to the HLIE regimen, after which calcium signals were recorded using a fiber photometry system (ThinkerTech Inc., China) in awake, freely moving mice. Fluorescence signals of GCaMP6s were emitted by 470 nm lasers and represented calcium activity in glutamatergic neurons. The data were exported as MATLAB files for further analysis. The fluorescence changes (ΔF/F%) were calculated as (F – F_0_)/(F_0_ – V_offset_), where F_0_ is the baseline fluorescence signal averaged over a 2‐s time window prior to the 120‐s acquisition period and V_offset_ is the fluorescence signal recorded before the cannula was connected to the optical fiber. ΔF/F% values are presented as heatmaps or average plots, with shaded areas indicating the standard error of the mean (SEM).

### 2.9 Statistical Analysis

All data are expressed as mean ± standard deviation (SD) with a sample size of 3–10 biological replicates per group. Data were analyzed using Prism 7 software (Graph Pad Software Inc., California, USA), SPSS (version 16.0, IBM), and Matlab R2017b (MathWorks, Massachusetts, USA). In the MWM study, the interaction between speed profile time and grouping factors during training was analyzed using a two‐way repeated measures analysis of variance (ANOVA), and between‐group comparisons of motor parameters obtained from probe tests were analyzed using a one‐way ANOVA with a Tukey's test as a post hoc analysis. The difference between the means of two independent groups was compared using an unpaired Student's *t*‐test. The Wilcoxon signed‐rank test was used for comparisons of the △F/F value, while an independent samples *t*‐test was used to analyze the Ca^2+^ signal data of area under the curve (AUC). *p* value < 0.05 was considered statistically significant.

## 3 Results

### 3.1 HLIE Induces Cognitive Dysfunciton in Mice

To assess the impact of HLIE on cognitive function in mice, we conducted a series of cognitive behavioral tests following a 7‐day HLIE regimen. These tests included the MWM, NOR, and Y maze (Figure 1A). As depicted in Figure 1B,C, there were no significant differences in body weight or food intake between the HLIE and Ctrl groups during the exercise period. In the spatial acquisition phase of the MWM, neither escape latency (Figure 1D) nor swimming speed (Figure 1E) showed significant differences between the groups. The heatmap of swimming tracks during the probe trial, conducted without the platform, is shown in Figure 1F. During the probe trial, mice in the HLIE group spent less time in the target quadrant and crossed the platform fewer times compared to the Ctrl group, with no differences in overall locomotor activity observed (Figure 1G–I). Additionally, spontaneous alternation behavior was assessed using the Y‐maze test to evaluate short‐term spatial memory (Figure S1A). The results showed that HLIE mice exhibited lower spontaneous alternation compared to the Ctrl group (Figure S1B–D). Similarly, in the NOR test, which assesses working memory, HLIE mice showed a reduced discrimination index (Figure S1E–H). Collectively, these findings suggest that HLIE may induce cognitive dysfunction in mice. Additionally, serum levels of LDH, CK, and UREA were significantly elevated in the HLIE group compared to the Ctrl group (Figure S1I–K), suggesting that HLIE exposure induced exercise‐related peripheral fatigue. HLIE animals exhibited significant corticosterone elevations (Figure S1L), confirming substantial psychological stress induction during forced treadmill running.

### 3.2 HLIE Alters Brain Regional Activity and Disturbs HPC‐mPFC Network Function in Mice

**FIGURE 2:**
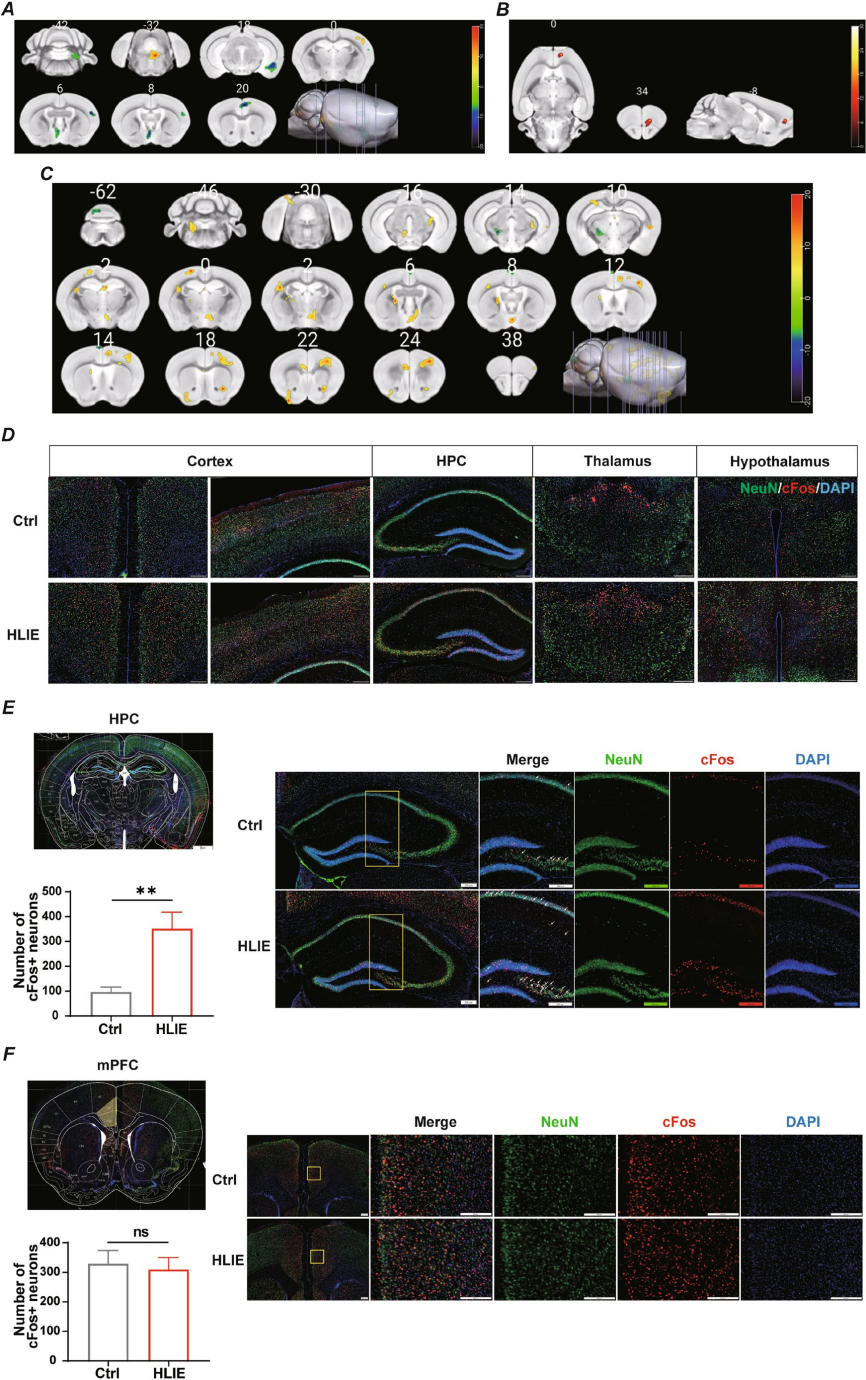
Alterations of network function and neural reactivity in the HPC and mPFC after 7‐day HLIE exposure. (A, B) Bold‐signal changes of rs‐fMRI in the HLIE group according to the results of the ReHo analysis (A) and ALFF analysis (B) compared with the Ctrl group. *n* = 3 mice per group. The voxel‐level height threshold was *p* < 0.005, and the cluster‐extent threshold was greater than 10 voxels. (C) Rs‐fMRI connectivity maps generated by correlation analysis of band‐pass filtered BOLD signals using a seed defined in the HPC. (D) Representative images of c‐Fos immunohistochemistry in regions of activation. Scale bar, 200 μm. (E, F) Merged fluorescence image of NeuN (green) co‐stained with c‐Fos (red) and DAPI (blue) in the HPC (E) and the mPFC (F) slices. The white arrowheads indicate the colocalized cells that expressed c‐Fos positive neurons. Scale bar, 200 μm. The number of c‐Fos positive neurons was measured with unpaired Student's *t*‐test (*n* = 3). Data are presented as mean ± SD. **p < 0.01.

To analyze the brain‐wide intrinsic functional response to HLIE, we conducted rs‐fMRI study in mice following HLIE exposure, utilizing regional homogeneity (ReHo) and amplitude of low‐frequency fluctuations (ALFF) analyses based on blood oxygen level dependent (BOLD) signal. In the ReHo analysis, widespread alterations in regional spontaneous activity were observed in HLIE mice compared to Ctrl mice (Figure 2A, Table S1). Notably, regions involved in motor coordination, such as the primary somatosensory cortex and the arbor vitae, exhibited significant activation. In contrast, HLIE mice showed decreased activity in the limbic system and neocortex, particularly in the anterior cingulate cortex (ACC), a region known to be anatomically subdivided from the mPFC [43]. In the ALFF analysis, an increased zALFF value was detected in the ventrolateral orbital cortex of HLIE mice compared to the Ctrl group (Figure 2B, Table S2). Table S1 and Table S2 detail the TMBA regions, including cluster sizes and peak T‐values for the differences in zReHo and zALFF values between groups, respectively. Notably, seed‐based analysis revealed significantly decreased functional connectivity from the HPC to the ACC in HLIE mice (Figure 2C, Table S3). These results suggest that HLIE leads to diminished activation in the mPFC and impaired functional connectivity within the HPC‐mPFC circuit, indicating potential dysfunction in this neural pathway.

To further identify specific responsive brain regions, we assessed whole‐brain c‐Fos immunoreactivity, which revealed a broad increase in c‐Fos signal in the cortex, HPC, thalamus and hypothalamus of HLIE mice (Figure 2D). Notably, robust c‐Fos activation was observed in the HPC of HLIE mice (Figure 2E), contrasting with no significant change in the mPFC (Figure 2F), establishing hippocampal hyperactivation as the core driver of network dysfunction.

### 3.3 HLIE Alters the Transcriptional Features in the HPC

**FIGURE 3:**
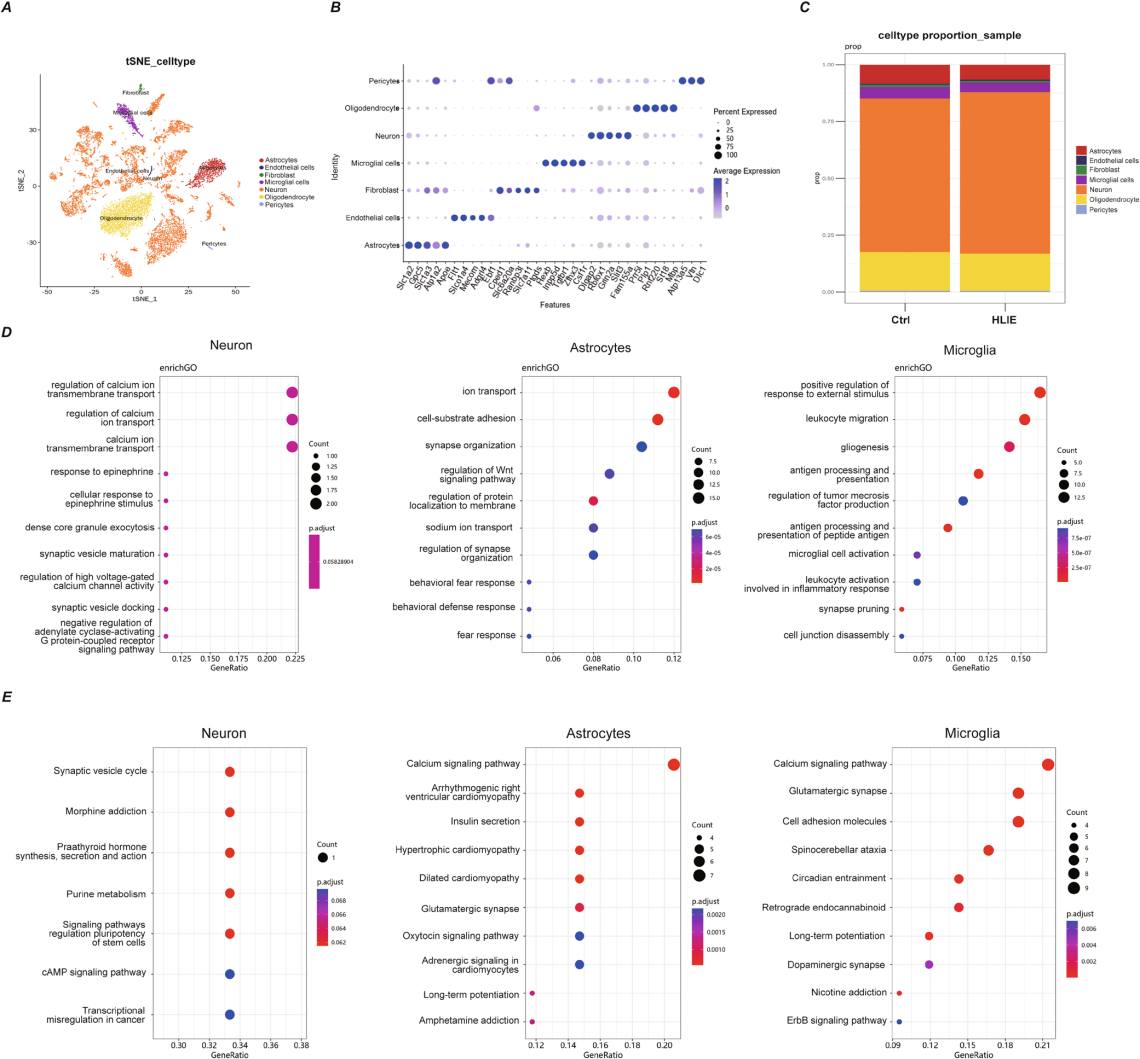
Single‐nucleus transcriptome in the HPC of HLIE mice. (A) t‐SNE plots display 7 identified cell types within the HPC. (B) Dot plots of the expression levels and the percentage of cells expressing markers for each cell type. (C) Proportions of each cell population in the grouped samples. (D) Top 10 GO enrichment pathways of the highly expressed genes in neurons, astrocytes, and microglia. (E) Top 10 KEGG enrichment pathways of the highly expressed genes in neurons, astrocytes, and microglia.

To characterize the molecular features of the hippocampus in HLIE mice, we performed snRNA‐seq analysis in the HPC. After filtering out low‐quality nuclei, we obtained sequencing data from 7307 cells in the Ctrl group and 10,552 cells in the HLIE group meeting quality control standards. These data were subjected to unsupervised clustering to elucidate cell‐type composition (Figure S2A,B). Key marker genes for each cell cluster are shown in Figure S2C. Subsequently, we identified seven major cell types based on established markers specific to each type, visualized using t‐distributed stochastic neighbor embedding (*t*‐SNE) (Figure 3A,B). Significant differences in the proportions of cell types were observed between the two groups (χ^2^ = 35.812, *p* < 0.001) (Figure 3C). Notably, the proportion of neurons in the HLIE group (71.1%) was higher than that in the Ctrl group (67.6%), whereas the proportions of astrocytes (8.4% in Ctrl vs. 6.5% in HLIE) and microglia (5.0% in Ctrl vs. 4.3% in HLIE) were decreased. These findings suggest that these three types of neural cells may play a role in the disruption of neural activity.

Next, differential gene expression analysis was performed for these three types of neural cells, identifying 43 differentially expressed genes (DEGs) in neurons, 215 in astrocytes, and 178 in microglia. We then performed enrichment analysis on these DEGs. Figure 3D,E shows the functional terms and pathways enriched through Gene Ontology (GO) and Kyoto Encyclopedia of Genes and Genomes (KEGG) analyses. Notably, in these three cell types with differing proportions, the DEGs were primarily enriched in processes such as the synaptic vesicle cycle, calcium signaling pathway, glutamatergic synapse, and long‐term potentiation, all of which are all recognized as biological foundations of cognitive function.

### 3.4 HLIE Induces Hyperexcitation of Hippocampal Glutamatergic Neurons in Mice

**FIGURE 4:**
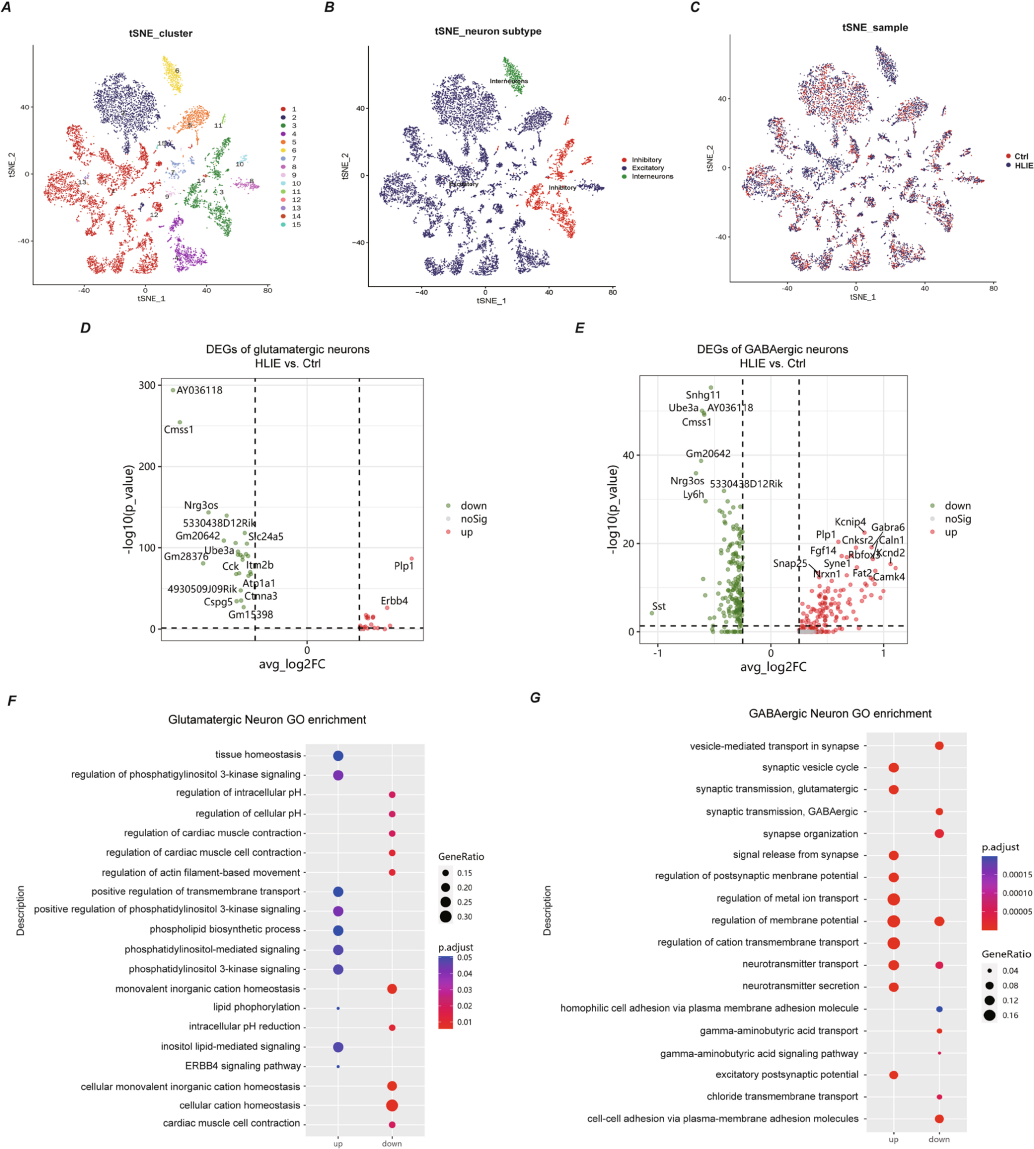
Transcriptome profiles of different neuronal subtypes within the HPC of HLIE mice. (A) t‐SNE plots display 45 identified subtypes of neurons within the HPC. (B) Annotated t‐SNE map of neuronal subtypes. (C) Distribution of samples on the t‐SNE plot by group. (D, E) Volcano plots of differentially expressed genes in the glutamatergic neurons (D) and GABAergic neurons (E) between the two groups. (F, G) Dot plots of GO functional enrichment analysis for differentially expressed genes in the glutamatergic neurons (F) and GABAergic neurons (G).

To further investigate the characteristics of hippocampal neurons in HLIE mice, we used a dimensionality reduction approach and identified a total of 15 clusters (Figure 4A). Based on well‐established cell markers, these clusters were classified into three subtypes: excitatory neurons, inhibitory neurons, and interneurons (Figure 4B). Cells were color‐coded based on their respective groups (Figure 4C). Glutamatergic neurons (Neu^Glu+^) are the predominant excitatory neurons in most brain regions, whereas inhibitory neurons and interneurons exhibit considerable heterogeneity across the brain, predominantly consisting of GABAergic neurons (Neu^GABA+^) in the HPC region [44]. The DEGs in Neu^Glu+^ and Neu^GABA+^ are shown in Figure 4D,E. GO analysis revealed a decline in functions related to cation homeostasis in Neu^Glu+^ neurons. In Neu^GABA+^ neurons, the significantly enriched biological processes of the DEGs primarily included synaptic vesicle cycling, synaptic organization, neurotransmitter secretion and transport, and regulation of synaptic membrane potential (Figure 4F,G).

**FIGURE 5:**
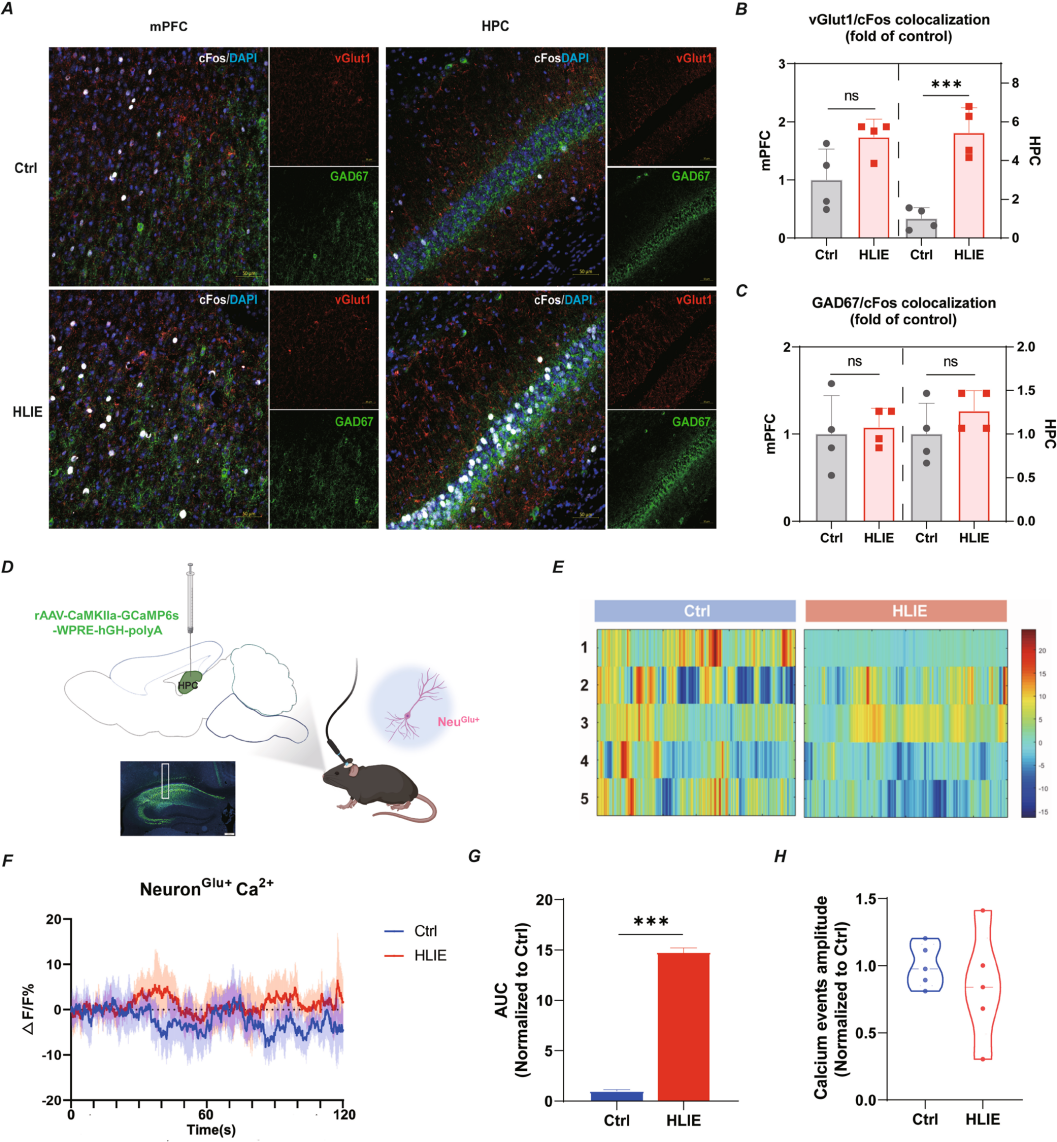
HLIE induces hyperactivation of glutamatergic neurons in the HPC. (A) Representative immunofluorescence images showing co‐staining of vGlut1 (red), GAD67 (green), and c‐Fos (white) in mouse mPFC and HPC slices. The blue staining indicates cell nuclei (DAPI); scale bar: 50 μm. (B) Statistical analysis of the number of vGlut1 and c‐Fos co‐stained cells. (C) Statistical analysis of the number of GAD67 and c‐Fos co‐stained cells (unpaired Student's *t*‐test. *p < 0.001; ns: Not significant; *n* = 4/group). (D) Schematic of experiment for Ca^2+^ signal recording of glutamatergic neurons in HPC. (E) Heatmaps of hippocampal Ca^2+^ activities of glutamatergic neurons after HLIE exposure for 120 s (*n* = 5). (F) Comparison of Ca^2+^ activity signals between Ctrl (blue) and HLIE group (red), expressed as ΔF/F (%). (G) The difference in the AUC of Ca^2+^ activity between Ctrl (blue) and HLIE group (red), ***p < 0.001. (H) The violin plots depicting the amplitude of calcium events of glutamatergic neurons in HPC show no significant difference between the two groups.

Next, immunofluorescence results revealed that c‐Fos was predominantly co‐localized with Neu^Glu+^, but not with Neu^GABA+^, in the HPC of HLIE mice. However, no significant differences were observed in these two types of neurons in the mPFC (Figure 5A–C). These findings suggest that HLIE is strongly associated with the activation of hippocampal glutamatergic neurons. To further investigate this, we used fiber photometry to measure the Ca^2+^ activity in hippocampal glutamatergic neurons (Figure 5D). A virus‐encoded Ca^2+^ sensor (rAAV‐CaMKIIa‐GCaMP6s) was injected, and an optical fiber was implanted at the HPC. Three weeks later, brain slices were imaged for green fluorescence to confirm successful virus infection and optic fiber location. At the end of the HLIE regimen, spontaneous Ca^2+^ events were recorded in both Ctrl and HLIE mice. The combined results demonstrated that HLIE led to a significant increase in spontaneous calcium signals (*Z* = −77.654, *p* < 0.05) (Figure 5E,F), as indicated by a lower AUC compared to the Ctrl group (Figure 5G), consistent with the observations in immunofluorescence staining. However, no significant differences in the amplitude of calcium events were detected (Figure 5H). Therefore, we conclude that HLIE induces excessive activation of glutamatergic neurons in the HPC, which may contribute to the dysfunction of the HPC‐mPFC circuit.

### 3.5 Chemogenetics Inhibition of HPC Glutamatergic Neurons Alleviates Cognitive Dysfunction Induced by HLIE

**FIGURE 6:**
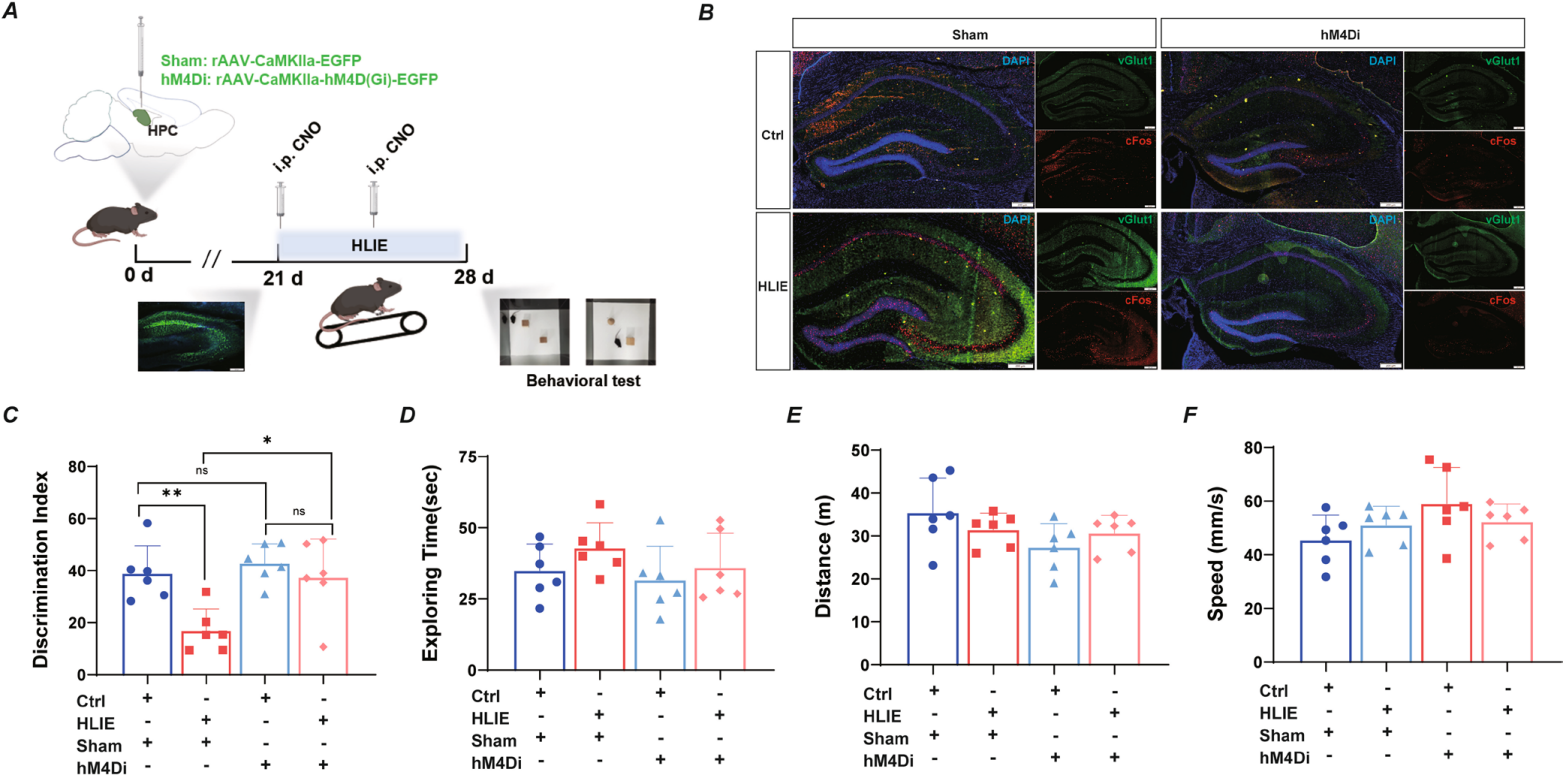
Chemogenetics inhibition of glutamatergic neurons in the HPC alleviated cognitive deficits induced by HLIE. (A) Schematic of the strategy to express hM4Di and (B) representative fluorescence images of hM4Di expression in the HPC. Scale bar: 200 μm. (C–F) Cognitive function was assessed by NOR test. HLIE‐induced cognitive deficits were improved, as suggested by the alterations in discrimination index (C) with no obvious changes in exploring time (D), activity distance (E), and speed (F). Data are presented as mean ± SD. *p < 0.05; **p < 0.01; ns, no significance.

To further investigate whether hyperactivated hippocampal glutamatergic neurons contribute to the cognitive deficits induced by HLIE, we expressed an inhibitory designer receptor or a control vector in the HPC via stereotactic injection, followed by a three‐week incubation period. We then adiministered the designer drug CNO during HLIE to activate the chemogenetic system (Figure 6A). Fluorescence image showed that vGlut1 immunoreactivity did not differ significantly between the Ctrl and HLIE groups in hM4Di mice, whereas vGlut1 levels were markedly increased in HLIE mice compared to Ctrl mice injected with Sham‐virus (Figure 6B). These results suggest that hM4Di expression effectively suppresses the activation of glutamatergic neurons induced by HLIE. Cognitive behavior was then assessed using the NOR test at the end of HLIE regimen. The results showed that virus injection did not significantly affect motor ability of the mice, as no statistical differences were observed in object exploration time, travel distance, or speed among all groups during the NOR test. However, the discrimination index was significantly lower in Sham mice after HLIE compared to sedentary controls, indicating that Sham virus injection did not prevent the HLIE‐induced impairment of working memory. In contrast, no significant difference in the discrimination index was observed between sedentary and HLIE groups in hM4Di mice (Figure 6C–F), suggesting that inhibition of hippocampal glutamatergic neurons partially alleviated the cognitive impairment induced by HLIE. Additionally, serological indicators of exercise fatigue showed no significant differences between the two virus injection groups, suggesting that peripheral fatigue was unaffected (Figure S3A–C). Together, these findings support the hypothesis that overactivation of hippocampal glutamatergic neurons contribute to HLIE‐induced cognitive dysfunction.

## 4 Discussion

It is well demonstrated that appropriate exercise could improve individuals' overall health and physical performance. Recently, the potential negative effects of excessive exercise on the body have attracted much attention, as certain specific groups of people experience excessive exercise in their occupational careers. For instance, we and others have previously shown that excessive exercise has negative effects on cardiac, hepatic, intestinal, and metabolic health [45, 46, 47, 48]. However, the effects of excessive exercise on cognitive function and its underlying mechanisms remain to be fully elucidated. In the present study, it was found that excessive exercise resulted in impaired cognitive function, evidenced by declines in a series of cognitive behavioral tests in mice. The dysregulation of hippocampal glutamatergic neurons induced by excessive exercise might be involved in this impaired cognitive function. These findings highlight hippocampal glutamatergic neurons as potential targets for the prevention of cognitive function impairment during excessive exercise.

In this study, a mouse model of excessive exercise was established using a 7‐day treadmill running protocol, in which mice ran at a speed of 25 m/min for 90 min per day for 7 consecutive days. This regimen was considered excessive exercise due to its high intensity, prolonged duration, and insufficient recovery during the training period. This high‐intensity endurance exercise model was based on previous studies [36, 37, 49]. In accordance with previous findings in both humans and rodents [13, 50], we found that mice exhibited impaired cognitive behaviors after HLIE exposure, accompanied by elevated serum markers of fatigue. Mechanistically, in addition to the previously reported findings, pathophysiological changes of central nervous system, which are closely related to cognitive impairment [20, 51, 52], we should also consider that neural networks, consisting of neural cells and circuits, might directly modulate neurobehaviors. From the results of rs‐fMRI analysis, several regions showing increased BOLD response were mainly involved in the modulation of brain functions such as motor coordination, behavioral state regulation, and emotional responses, which aligns with physiological fluctuations related to motor activity induced by HLIE. By contrast, spontaneous activity in several brain regions belonging to the limbic system and neocortex was markedly suppressed, including ACC, an important substructure of the mPFC. In line with our findings, negative BOLD responses in the PFC have been observed in cycling exercise participants undergoing high‐intensity exercise [19]. In HPC‐based functional connectivity analysis, enhanced connectivity was observed in circuits related to sensorimotor system. Importantly, the functional connectivity between the HPC and mPFC was suppressed by HLIE, suggesting the diminished modulation of cognitive functions such as learning and memory [23]. However, we explicitly acknowledge that the small sample size (*n* = 3 per group) in our fMRI analysis constitutes a technical limitation of this study. While consistent with precedent literature for fMRI investigations [53], this constrained cohort size limits our ability to perform complex subgroup analyses or account for potential biological variability across heterogeneous populations. Considering the need to minimize motion‐related artifacts and ensure stable image acquisition, the fMRI data were collected under anesthesia, future studies are required to uncover the imaging features of neural activity in task states. These findings represent the neurobiological basis for functional impairments in the HPC‐mPFC circuit. Furthermore, our study reveals that among several specifically activated brain regions in whole‐brain c‐Fos immunoreactivity after HLIE, the cortex, including motor cortex and somatosensory cortex, showed activation, which might reflect adaptive changes induced by exercise. In addition, the activation of relay nuclei, such as the thalamus and the hypothalamus, is implicated in the robust regulation of body temperature and energy metabolism under exercise conditions [54, 55, 56]. Notably, the HPC is the only region that has been clearly linked to cognitive function. Thus, we confirm that the HPC might be the core region affected by HLIE, contributing to the cognitive impairment.

snRNA‐seq has emerged as a powerful and efficient tool for determining cellular heterogeneity in both physiological and disease conditions [57, 58]. In this study, we performed snRNA‐seq method for the first time to profile cellular transcriptional features of the HPC in HLIE model. The changed proportions of neurons and glia cells may reflect cellular response or adaption following metabolic stress. Specifically, the increase in neuronal proportion is quantitatively consistent with exercise‐induced hippocampal neurogenesis [59]. The decreased astrocyte and microglial proportions likely reflect activity‐dependent glial adaptations to sustained metabolic stress, wherein acute stress affects distinct facets of astrocyte morphology, calcium signaling, and intercellular coupling [60], while the loss of hippocampal microglia was confirmed in depression induced by chronic stress [61]. Analysis of transcriptional features revealed that HLIE significantly impacts biological processes related to cognitive functions, such as synaptic vesicle cycling, calcium signaling pathways, glutamatergic synapses, and long‐term potentiation. Notably, subpopulation analysis of neurons revealed that HLIE significantly downregulated genes associated with cellular pH regulation and cation homeostasis in glutamatergic neurons. This suggests an imbalance in ion homeostasis of hippocampal glutamatergic neurons, which may impair normal action potentials and signal transmission. However, the deviation of heterogeneity during sample collection and preparation cannot be completely ruled out. Therefore, these results should be interpreted with caution as they provide important and novel biological insights, but further investigation is required. Remarkably, a significant increase in c‐Fos expression was observed in glutamatergic neurons in the HPC of HLIE mice compared to Ctrl mice, aligning with hyperactivation of calcium signals. Moreover, targeted chemogenetic inhibition of glutamatergic neurons in the HPC alleviated HLIE‐induced cognitive impairment, implying that hyperactivation of hippocampal glutamatergic neurons plays a crucial role in modulating cognitive deficits associated with HLIE. Therefore, we propose that the identified cellular abnormalities may resemble the hyperactivity of hippocampal pyramidal neurons observed in conditions such as Alzheimer's disease (AD) or epilepsy, potentially serving as a key initiating factor in cognitive dysfunction [62, 63]. Our findings provide a mechanistic framework for understanding cognitive fatigue phenomena in human populations undergoing sustained physical exertion. Strenuous exercise induces transient elevations in glutamate/glutamine (Glx) in several brain regions—positively correlating with working memory enhancement in the immediate post‐exercise phase [64]. As excessive accumulation of glutamate exhibits neurotoxicity, the hippocampal glutamatergic hyperactivity observed in HLIE mice translational alignment reveals a critical exposure‐duration threshold: transient glutamate flux enhances cognition, whereas chronic excess precipitates excitotoxic neuronal apoptosis. Critically, the basic research on this excitotoxic mechanism has practical significance for the clinical translation of cognitive protection strategies for the occupational cohorts subjected high‐intensity training—particularly elite athletes and military personnel during sustained operations.

However, there are some limitations to the present study. HLIE regimen was inevitably accompanied by some non‐specific stressors, such as electroshocks, as well as psychological stressors including fear and anxiety. The present study considered HLIE and non‐specific stressors as a combined stimulus affecting cognitive function. Future studies should further measure and consider stress levels to better account for these effects. In addition, given the gender differences in motor ability and cognitive processes, which are rooted in structural and functional differences in the brain, further studies are needed to explore the impact of HLIE on cognitive function in female animals.

## 5 Conclusions

Our present study demonstrates that the cognitive deficits following HLIE are primarily driven by the hyperactivation of hippocampal glutamatergic neurons, which subsequently disrupts the functional integrity of the HPC‐mPFC circuits. These findings provide novel mechanistic insights into the cognitive dysfunction associated with excessive exercise and offer a foundation for developing strategies to maintain or enhance cognitive function in scenarios where excessive exercise is unavoidable. Specifically, real‐time neurometabolic biomarkers and astrocytic glutamate regulators emerge as promising countermeasures against exercise‐induced cognitive impairment.

## Author Contributions

Qian Bai performed the experiments, analyzed the data, and drafted the manuscript; Le Wang, Hongtao Yu and Xiaolei Wang collected samples and participated in the experiments. Hedong Lang and Jian Wang aided in methodology, data analysis and manuscript editing. Jundong Zhu aided in formal analysis and manuscript review. Ka Chen designed and administrated the project, aided in original draft preparation and editing. Mantian Mi designed and administrated the project, acquired funding, and finalized the manuscript.

## Funding

This research was funded by Scientific Research Grant, grant number No. ALJ22J003.

## Ethics Statement

All animal experiments were conducted in accordance with the National Institutes of Health Guide for the Care and Use of Laboratory Animals and were approved by the Animal Ethics Committee of the Army Medical University (AMUWEC2020728).

## Consent

The authors have nothing to report.

## Conflicts of Interest

The authors declare no conflicts of interest.

## Supporting information


**Figure S1:** HLIE exposure induced cognitive underperformance of mice in the NOR and Y maze tests and peripheral fatigue.

**Figure S2:** Single‐nuleus profile of HPC isolated from Ctrl mice and HLIE mice.

**Figure S3:** The impact of chemogenetic manipulation on the serological indicators related to exercise fatigue and hippocampal morphology.

**Method and Materiel S1.** Detailed protocols of behavioral tests.

**Method and Materiel S2.** Detailed protocols of snRNA‐Seq analysis.

**Table S1:** Brain regions with ReHo differences in mice after 7‐day HLIE exposure.

**Table S2:** Brain region with ALFF differences in mice after 7‐day HLIE exposure.

**Table S3:** Brain regions with seed‐based functional connectivity differences in mice after 7‐day HLIE exposure.

## Data Availability

The data that support the findings of this study are available from the corresponding author upon reasonable request.
