# Supplementary figures and images for: Hippocampal Glutamatergic Hyperactivation Mediates High‐Loading Intensity of Exercise‐Induced Cognitive Deficits Via HPC‐mPFC Circuit Dysfunction
Source: CNS Neurosci Ther. 2026 Jun 18;32(6):e70928. doi: 10.1002/cns.70928 (PMC13278025; doi:10.1002/cns.70928)

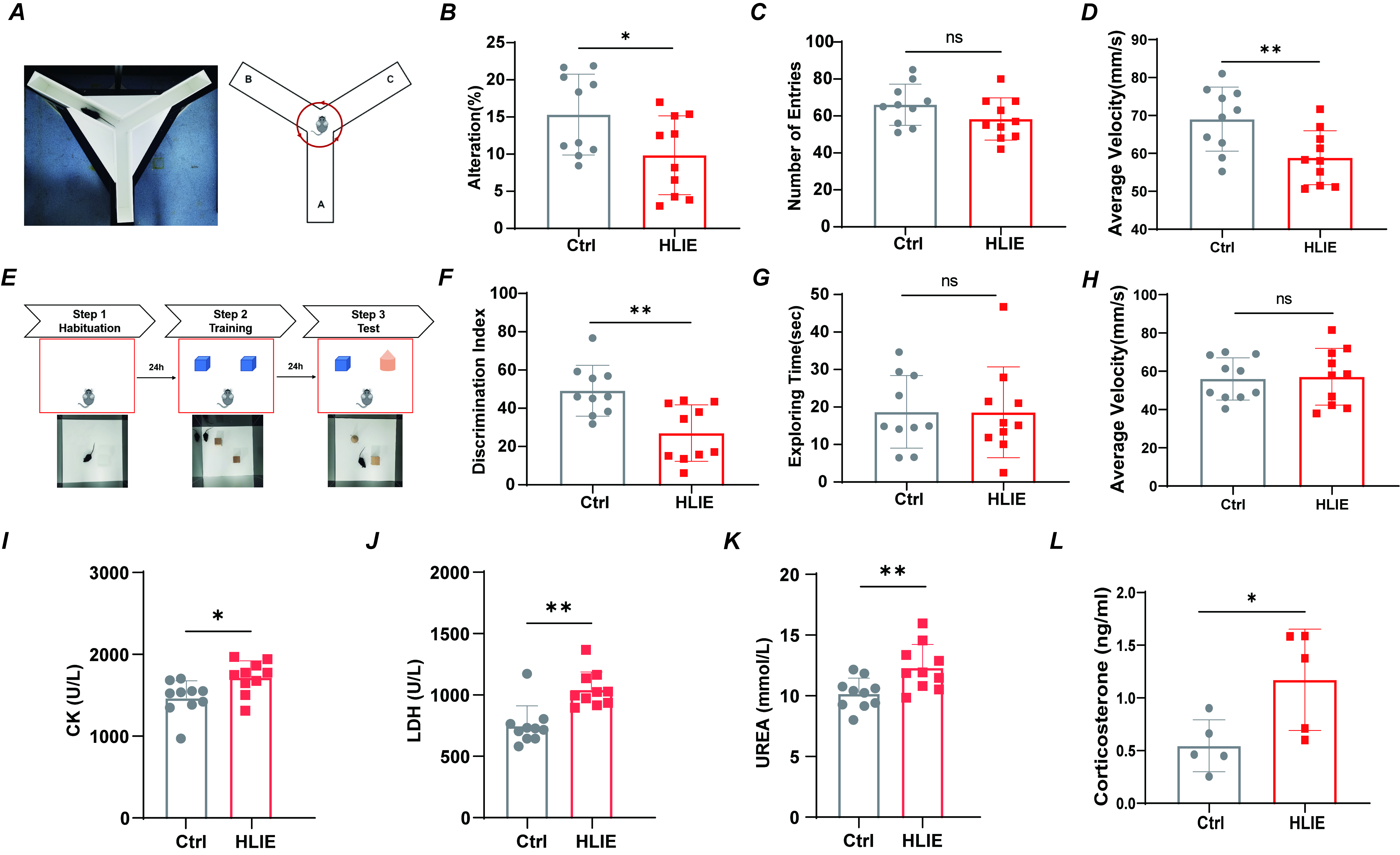

Supplement: Supplementary file 1 — Figure S1: HLIE exposure induced cognitive underperformance of mice in the NOR and Y maze tests and peripheral fatigue. [file CNS-32-e70928-s001.tif]

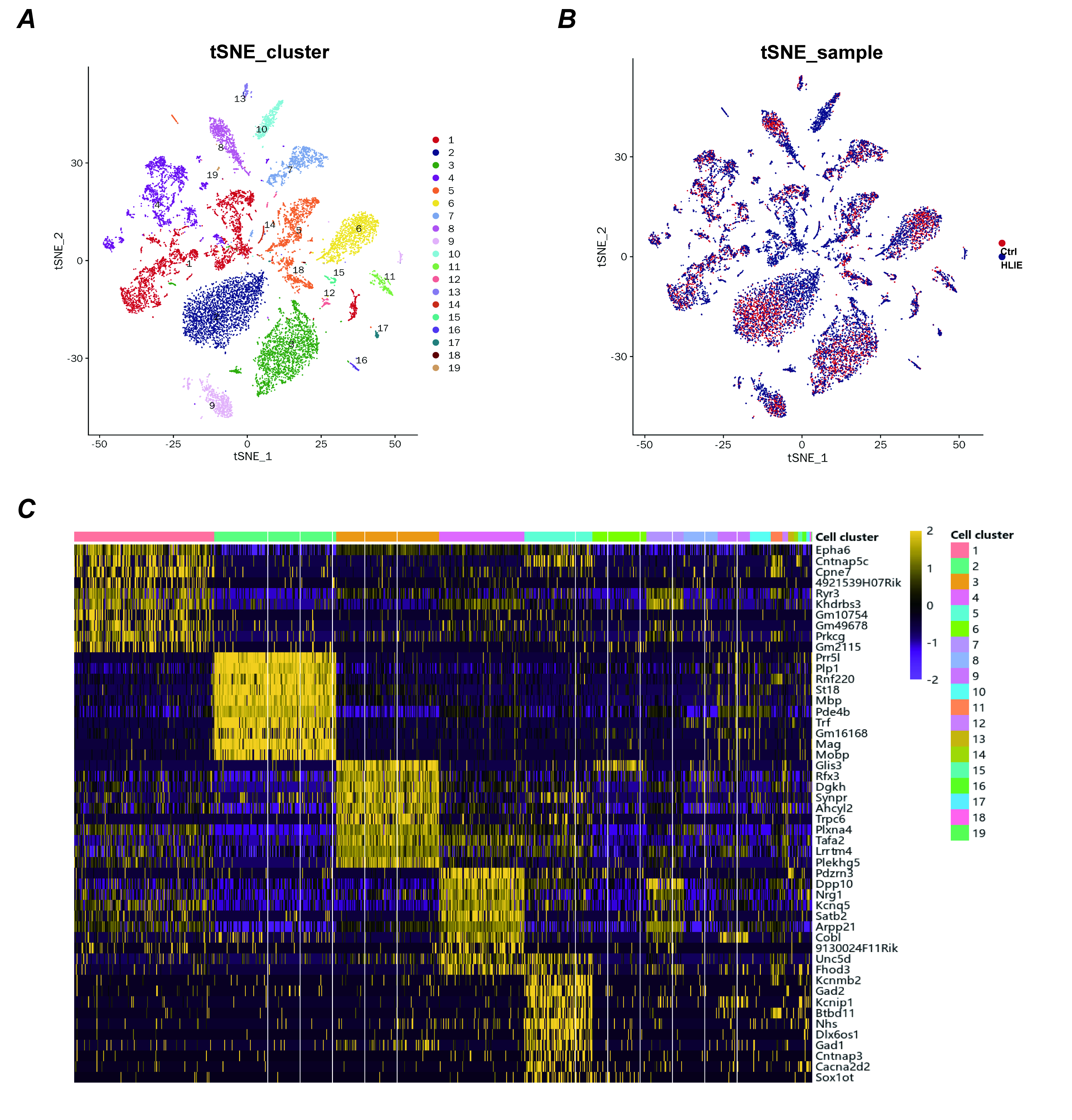

Supplement: Supplementary file 2 — Figure S2: Single‐nuleus profile of HPC isolated from Ctrl mice and HLIE mice. [file CNS-32-e70928-s004.tif]

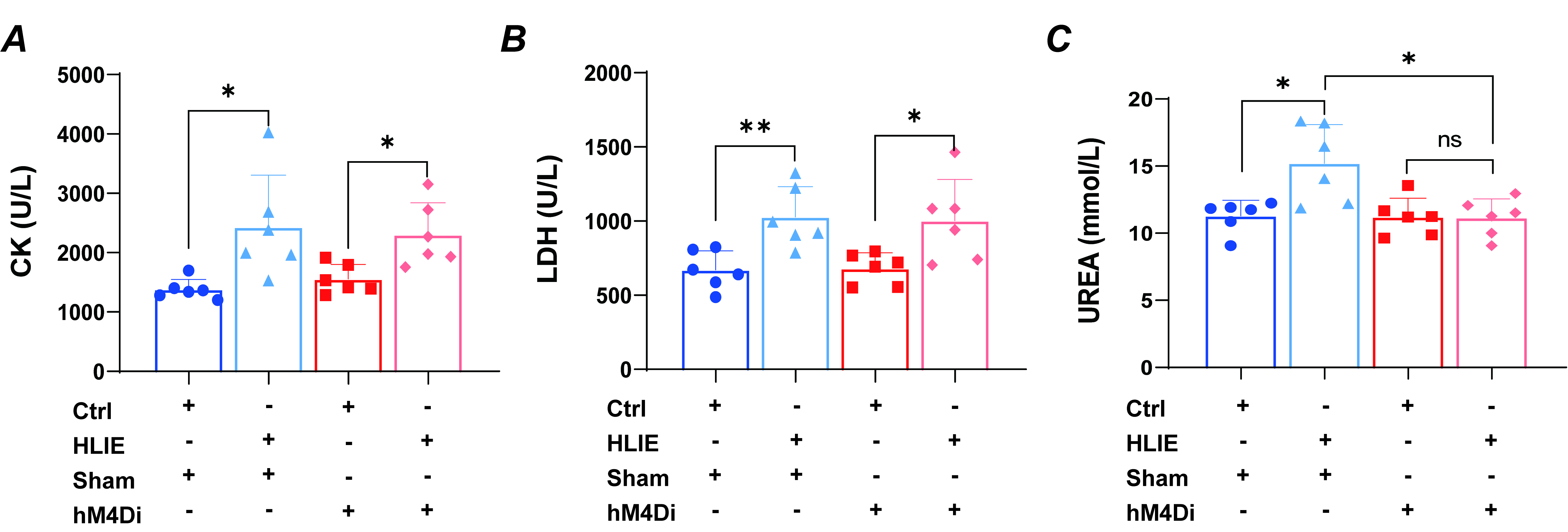

Supplement: Supplementary file 3 — Figure S3: The impact of chemogenetic manipulation on the serological indicators related to exercise fatigue and hippocampal morphology. [file CNS-32-e70928-s006.tif]
