# Supplementary material for: Hippocampal Glutamatergic Hyperactivation Mediates High‐Loading Intensity of Exercise‐Induced Cognitive Deficits Via HPC‐mPFC Circuit Dysfunction
Source: CNS Neurosci Ther. 2026 Jun 18;32(6):e70928. doi: 10.1002/cns.70928 (PMC13278025; doi:10.1002/cns.70928)
Supplement: Supplementary file 4 — Method and Materiel S1. Detailed protocols of behavioral tests. [file CNS-32-e70928-s005.docx]

**Method and Materiel S1.** Detailed protocols of behavioral tests

*Morris Water Maze (MWM)*

The MWM test is one of the most well-established and classic tests of cognitive spatial ability in rodents. As previously described, in the spatial acquisition phase, mice were trained to locate a 12-cm plexiglass platform submerged 1.5-cm below the water. The water was made opaque by adding skim milk powder. Mice were trained four times per day, starting from each of the four quadrants, for 5 consecutive days. The total distance traveled, average speed, and latency to locate the platform for each trial were recorded. Data from the four daily trials were averaged for statistical analysis. A probe trial was performed immediately on the same day after the HLIE procedure. During this 60-second trial, the platform was removed. The time spent in the target quadrant, platform crossings, and average swimming speeds were determined from videotape recordings.

*Novel Object Recognition (NOR)*

The NOR test has been widely used to investigate learning and memory in mice. As previous studies described, (1) Mice were allowed to freely explore the empty open-field arena (40-cm length × 40-cm width × 40-cm height) for 10 minutes. (2) After 24 hours, two identical objects (cube building block, 5cm×5cm×5cm) were placed at the far ends of the diagonal line of the arena. The mice were then placed in the arena, facing away from the objects. (3) After a 24-hour familiarization period, one of the two familiar objects was replaced with a novel object (cone-shaped building block, bottom diameter=5 cm) at the same location. The mice were then placed in the apparatus and allowed to explore freely. The time, frequency, and distance of each mouse's exploration of the objects were recorded using the VisuTrack system (XR-VT, Xinruan, China) for 10 minutes. The discrimination index (DI) was calculated using the following formula: DI = New object exploration time/(New object exploration time + Familiar object exploration time).

*Y Maze*

The Y-maze was used to assess the spatial working and reference memory of mice as described previously. Mice were placed at the center of the maze and allowed to freely explore the three arms. The number of arm entries and alternation triads are recorded using the VisuTrack system (XR-VT, Xinruan, China) over an 8-minute period to calculate the percentage of alternation: %Alternation = (Number of alternations/[Total number of arm entries-2]) × 100.
