# Supplementary material for: Hippocampal Glutamatergic Hyperactivation Mediates High‐Loading Intensity of Exercise‐Induced Cognitive Deficits Via HPC‐mPFC Circuit Dysfunction
Source: CNS Neurosci Ther. 2026 Jun 18;32(6):e70928. doi: 10.1002/cns.70928 (PMC13278025; doi:10.1002/cns.70928)
Supplement: Supplementary file 5 — Method and Materiel S2. Detailed protocols of snRNA‐Seq analysis. [file CNS-32-e70928-s002.docx]

**Method and Materiel S2.** Detailed protocols of snRNA-Seq analysis

Nucleus Isolation

Frozen HPC tissues from each group (n=3/group) were used for nucleus isolation. Briefly, the tissues were minced into 1 mm³ fragments and suspended in a digestion buffer containing collagenase type I (2 mg/ml) and DNase I (200 U/ml), then incubated in a water bath at 37°C for 45 minutes with gentle shaking in DMEM medium. The resulting solution was filtered through a 100 μm filter and centrifuged 400 g for 10 minutes at 4°C. The cell pellet was resuspended in red blood cell lysis buffer, incubated for 2 minutes, filtered through a 40 μm filter and centrifuged again 400 g for 10 minutes at 4°C. The final pellet was resuspended in PBS containing 0.04% BSA. The cell suspension was gently mixed by pipetting, and 10 μl of the cell suspension was mixed with 10 μl of AOPI dye. The mixture was transferred to a Countstar cell counting plate and allowed to settle for 30 seconds to 1 minute. The counting plate was inserted into the Countstar cell counter to perform manual quality control and cell counting. Typically, the cell count should be ≥ 50,000, and the cell viability should be ≥ 80%. The qualified cells were washed and resuspended to achieve a concentration of 700–1200 cells/μl for loading onto the 10x Genomics Chromium™ system.

Single-Nuclei RNA Sequencing

Single-cell 3' gene expression profiling was performed using the Chromium Next GEM Single Cell 3’ Kit v3.1 and the Chromium Next GEM Chip G Single Cell Kit. The cell suspension was loaded onto the 10x Genomics Chromium Single Cell Controller to generate single-cell gel beads-in-emulsion (GEMs). Captured cells were lysed, and single-cell tagging was encoded by adding achieved by incorporating unique molecular identifiers (UMIs) during the reverse transcription within individual GEMs. The GEMs were subjected to oil phase treatment, and single-stranded cDNA was purified and enriched using magnetic beads, followed by cDNA amplification and quality control. The quality-checked cDNA was used to construct a next-generation sequencing library, through fragmentation, adapter ligation, and sample index PCR, followed by quantitative quality control of the library. Sequencing was performed on the Illumina NovaSeq 6000 platform (Illumina, San Diego, CA, USA) using the PE150 mode, achieving a sequencing depth of over 50,000 reads per cell.

Statistical Analysis of snRNA-Seq

Quality control analysis of the raw data was performed using FastQC (v0.11.9). The raw data were processed using Cell Ranger v7.0 to generate a filtered gene expression matrix. Data quality control and normalization were performed using Seurat v4.0.6, filtering out barcodes with fewer than 200 genes, more than 10% of the maximum UMIs, and cells with over 30% mitochondrial gene content. For each dataset, UMI counts were log-normalized using the Normalize Data function in Seurat v4.0.6. To identify highly variable genes, 2000 genes were selected using the Find Variable Features function. The expression levels of these 2000 genes were scaled and centered using the Scale Data function. Principal component analysis (PCA) was performed on the selected 2000 genes using Seurat, followed by cell integration across samples using the harmony algorithm (v0.1.1). After integration, the top 30 principal components were used for nonlinear dimensionality reduction with Uniform Manifold Approximation and Projection (UMAP) and t-Distributed Stochastic Neighbor Embedding (t-SNE). Initial cell subtype annotation was performed using SingleR (v1.8.0). Typical markers were applied for validation, and the results were further refined.

Enrichment Analysis

Differentially expressed genes (DEGs) for each cluster were identified using the FindAllMarkers function Seurat with default parameters. DEGs were classified as upregulated or downregulated based on │log2 fold change (FC)│ > 0.25. Gene Ontology (GO) and Kyoto Encyclopedia of Genes and Genomes (KEGG) pathway enrichment analyses were performed for upregulated and downregulated DEGs using the clusterProfiler package (v4.2.2). To facilitate enrichment analysis, gene symbols were converted to Entrez gene IDs using the bitr function in clusterProfiler. The p-values were adjusted using the false discovery rate (FDR) correction method, with a q-value threshold of 0.25.
