# Supplementary material for: Hippocampal Glutamatergic Hyperactivation Mediates High‐Loading Intensity of Exercise‐Induced Cognitive Deficits Via HPC‐mPFC Circuit Dysfunction
Source: CNS Neurosci Ther. 2026 Jun 18;32(6):e70928. doi: 10.1002/cns.70928 (PMC13278025; doi:10.1002/cns.70928)
Supplement: Supplementary file 6 — Table S1: Brain regions with ReHo differences in mice after 7‐day HLIE exposure. [file CNS-32-e70928-s003.docx]

**Table S1.** Brain regions with ReHo differences in mice after 7-day HLIE exposure

| Brain regions | Cluster size | Peak T value | TMBA template coordinates | | |
| --- | --- | --- | --- | --- | --- |
|  |  |  | X | Y | Z |
| Medial_preoptic_nucleus_Left | 26 | -16.0473 | 2 | 8 | -14 |
| Basolateral_amygdalar_nucleus/_posterior_part_Right | 45 | -17.9094 | -30 | -18 | -10 |
| Superior_central_nucleus_raphe_Left | 58 | 21.9144 | -4 | -32 | 0 |
| Supplemental_somatosensory_area/_layer_6a_Right | 42 | -32.8347 | -32 | 6 | 6 |
| Arbor_vitae_Left | 10 | -7.8707 | -16 | -42 | 0 |
| Primary_somatosensory_area/_barrel_field/_layer_5_Right | 12 | 14.3321 | -24 | 0 | 18 |
| Anterior_cingulate_area/_dorsal_part/_layer_1_Left | 14 | -24.6164 | -2 | 20 | 14 |
