# Supplementary material for: Hippocampal Glutamatergic Hyperactivation Mediates High‐Loading Intensity of Exercise‐Induced Cognitive Deficits Via HPC‐mPFC Circuit Dysfunction
Source: CNS Neurosci Ther. 2026 Jun 18;32(6):e70928. doi: 10.1002/cns.70928 (PMC13278025; doi:10.1002/cns.70928)
Supplement: Supplementary file 7 — Table S2: Brain region with ALFF differences in mice after 7‐day HLIE exposure. [file CNS-32-e70928-s008.docx]

**Table S2.** Brain region with ALFF differences in mice after 7-day HLIE exposure

| Brain regions | Cluster size | Peak T value | TMBA template coordinates | | |
| --- | --- | --- | --- | --- | --- |
|  |  |  | X | Y | Z |
| Orbital_area/_ventrolateral_part/_layer_1_Right | 15 | 28.749 | -8 | 34 | 0 |
