# Supplementary material for: Hippocampal Glutamatergic Hyperactivation Mediates High‐Loading Intensity of Exercise‐Induced Cognitive Deficits Via HPC‐mPFC Circuit Dysfunction
Source: CNS Neurosci Ther. 2026 Jun 18;32(6):e70928. doi: 10.1002/cns.70928 (PMC13278025; doi:10.1002/cns.70928)
Supplement: Supplementary file 8 — Table S3: Brain regions with seed‐based functional connectivity differences in mice after 7‐day HLIE exposure. [file CNS-32-e70928-s007.docx]

**Table S3.** Brain regions with seed-based functional connectivity differences in mice after 7-day HLIE exposure

| Brain regions | Cluster size | Peak T value | TMBA template coordinates | | |
| --- | --- | --- | --- | --- | --- |
|  |  |  | X | Y | Z |
| Olfactory_tubercle_Left | 31 | 23.4808 | 18 | 22 | -24 |
| Anterior_hypothalamic_nucleus_Right | 52 | 20.25 | 0 | 8 | -18 |
| Caudoputamen_Right | 16 | 23.0942 | -16 | 18 | -14 |
| Ventral_tegmental_area_Left | 15 | 14.2156 | 6 | -16 | -8 |
| Zona_incerta_Left | 17 | -15.2766 | 14 | -14 | -6 |
| Medial_vestibular_nucleus_Left | 22 | 11.0856 | 12 | -46 | -4 |
| Secondary_motor_area/_layer_2/3_Right | 10 | 9.3098 | -20 | 38 | 4 |
| Caudoputamen_Left | 25 | 24.9234 | 14 | 6 | -2 |
| Orbital_area/_ventrolateral_part/_layer_2/3_Left | 22 | 18.2294 | 6 | 24 | 4 |
| Medial_geniculate_complex/_medial_part_Right | 13 | 10.8229 | -18 | -16 | 2 |
| Caudoputamen_Right | 10 | 18.3521 | -32 | -10 | -2 |
| Corpus_callosum/_body_Left | 43 | 20.1891 | 26 | 2 | 10 |
| Medial_habenula_Left | 11 | 21.7679 | -2 | -2 | 10 |
| Primary_motor_area/_Layer_4/5_Right | 115 | 26.3153 | -22 | 12 | 14 |
| Pyramus_(VIII)_Left | 11 | -13.4551 | 4 | -62 | 10 |
| Field_CA1_Left | 26 | 25.1719 | 14 | 0 | 24 |
| Anterior_cingulate_area/_dorsal_part/_layer_5_Right | 14 | 16.7349 | -4 | 14 | 16 |
| Retrosplenial_area/_dorsal_part/_layer_5_Left | 17 | 17.4994 | 20 | -30 | 24 |
| Anterior_cingulate_area/_dorsal_part/_layer_1_Left | 10 | -17.6222 | 4 | 14 | 22 |
